# Lead and Chromium Adsorption from Water using L-Cysteine Functionalized Magnetite (Fe_3_O_4_) Nanoparticles

**DOI:** 10.1038/s41598-017-03380-x

**Published:** 2017-08-09

**Authors:** Yana Bagbi, Ankur Sarswat, Dinesh Mohan, Arvind Pandey, Pratima R. Solanki

**Affiliations:** 1 Special Centre for Nanoscience, Jawaharlal Nehru University, New Delhi, 110067 India; 2 School of Environmental Sciences, Jawaharlal Nehru University, New Delhi, 110067 India; 3 Department of Physics, North Eastern Regional Institute of Science and Technology, Nirjuli, Arunachal Pradesh 791109 India

## Abstract

L-Cysteine functionalized magnetite nanoparticles (L-Cyst-Fe_3_O_4_ NPs) were synthesized by chemical co-precipitation using Fe^2+^ and Fe^3+^ as iron precursors, sodium hydroxide as a base and L-Cysteine as functionalized agent. The structural and morphological studies were carried out using X-ray powder diffraction, transmission electron microscopy, dynamic light scattering, scanning electron microscopy and energy dispersive X-ray spectroscopy, Fourier transform infrared spectroscopy, and UV-Vis spectrophotometric techniques. The zeta potential of bare Fe_3_O_4_ and functionalized L-Cyst-Fe_3_O_4_ NPs were +28 mV and −30.2 mV (pH 7.0), respectively. The positive surface charge changes to negative imply the presence of L-Cyst monolayer at particle interface. Band gap energy of 2.12 eV [bare Fe_3_O_4_NPs] and 1.4 eV [L-Cyst-Fe_3_O_4_ NPs] were obtained. Lead and chromium removal were investigated at different initial pHs, contact time, temperatures and adsorbate-adsorbent concentrations. Maximum Cr^6+^ and Pb^2+^ removal occurred at pH 2.0 and 6.0, respectively. Sorption dynamics data were best described by pseudo-second order rate equation. Pb^2+^ and Cr^6+^ sorption equilibrium data were best fitted to Langmuir equation. Langmuir adsorption capacities of 18.8 mg/g (Pb^2+^) and 34.5 mg/g (Cr^6+^) at 45 °C were obtained. Regeneration of exhausted L-Cyst-Fe_3_O_4_ NPs and recovery of Pb^2+^/Cr^6+^ were demonstrated using 0.01 M HNO_3_ and NaOH. L-Cyst-Fe_3_O_4_ NPs stability and reusability were also demonstrated.

## Introduction

Heavy metals’ presence above their prescribed limits in water bodies imprints toxic effects to the aquatic life and human health^1, 2^. Mining, agricultural and technological applications are common anthropogenic inputs of heavy metals exposure to human beings^2, 3^.

Lead and chromium are commonly used heavy metals associated with toxic poisoning even at low concentrations and bear no biological benefit to humans^1, 3^. Common sources of lead include effluents from battery manufacturing, steel industries, painting pigment, fuels, photographic materials, aeronautical, automobile, explosive manufacturing, and coating industries^4, 5^. Accumulation of lead in humans can cause cancer, kidney diseases, memory problems and high blood pressure, premature birth, brain damage, hearing loss, learning disabilities and a lower IQ level in children^6–8^.

Chromium is a toxic heavy metal and exists mainly in two oxidation states in water i.e. Cr (III) and Cr (VI)^9^. Cr (VI) species is more toxic than Cr (III). Cr (VI) a toxic, carcinogenic and highly soluble in aqueous systems^10^. Electroplating, leather tanning, mining, metal processing and film processing are common anthropogenic sources of chromium introduce to water bodies^9, 10^. The World Health Organization (WHO) recommends a permissible limit of 0.1 mg/L and 0.05 mg/L for lead and chromium, respectively^9^. The US Environmental Protection Agency (USEPA) recommends a maximum permissible limit of 0.1 mg/L and 0.015 mg/L for lead and chromium in drinking water, respectively.

Common methods employed for aqueous lead and chromium removal include electrochemical technique, reverse osmosis, membrane filtration, ion-exchange and adsorption^11–14^. Adsorption is a simple, economical and effective method for aqueous contaminants removal^15^. Adsorbents including activated carbons^15^, clay minerals^16^ and chitosan/natural zeolites^17^ have been explored for heavy metals’ aqueous remediation. However, their separation post-adsorption requires tedious filtration step.

To overcome this filtration, use of magnetite nanoparticles is a viable approach. Magnetite nanoparticles are super paramagnetic, hydrophilic and possess high surface area. Therefore, the exhausted nanoparticles can be stripped-out from aqueous systems using a simple magnet^18, 19^. Magnetite NPs aggregate due to long range Van der Waals’ forces, thereby difficult to stabilize their colloidal dispersions. As a result, there is a decrease in its surface area and adsorption capacity^20^. Thus, magnetite dispersion is a vital factor while considering its sorptive applications. Magnetite NPs have been modified using amine^21^, thiol^22^, naphthalimide^23^, and reactive blue-19^24^ to enhance the dispersion.

L-Cysteine (L-Cyst) [HO_2_CCH (NH_2_) CH_2_SH] is an amino acid with a redox and catalytic behavior. L-Cysteine also possesses remarkable metal binding properties^25^. It is a sulfur-containing amino acid having three functional groups (-SH, -NH_2_, -COOH), which has strong tendency to co-ordinate with cations including heavy metal ions^25, 26^. Sulfur in the -SH is divalent and endows L-Cyst with an additional function, acting as an antioxidant^26^. The -NH_2_ and -COOH functional groups in L-Cyst provides magnetite NPs a good bio-compatibility and solubility^27^. The -NH_2_ and -COOH groups in magnetite NPs participated well in the aqueous removal of Cu (II), Co (II), Ni (II), Zn (II), Pb (II), Cr (VI) and Cd (II) by forming various complexes^27, 28^. Many studies have reported enhancing biocompatible behavior of cysteine as well as incorporating antibacterial characteristics^29, 30^. Cysteine on one side induces biocompatibility for mammalian cell line while on other side promotes antibacterial behavior against bacterial strains.

In this paper, L-Cyst functionalized magnetite NPs were synthesized, characterized and successfully applied for aqueous Pb^2+^ and Cr^6+^ removal. Pb^2+^ and Cr^6+^ adsorption on L-Cyst-Fe_3_O_4_ NPs were performed at different initial pHs, temperatures, contact times and adsorbate-adsorbent concentrations. The sorption data were fitted to Freundlich and Langmuir isotherm equations. Sorption thermodynamic parameters were also determined. Pseudo-first and second order rate equations were used to fit the kinetic data.

## Experimental

### Materials and Methods

All the chemicals used were either AR or GR grades. Lead nitrate, Pb (NO_3_)_2_ (99%), potassium dichromate, K_2_Cr_2_O_7_ (99%), ethanol (99%), hydrochloric acid, HCl and sodium hydroxide, NaOH (98%) were obtained from Merck, India. Ferric chloride hexahydrate, FeCl_3_ . 6H_2_O (97%) and ferrous chloride tetra hydrate, FeCl_2_ . 4H_2_O (97%) were purchased from CDH, India. L-Cysteine (L-Cyst 98%) was purchased from Sigma Aldrich. Chemicals including Roswell Park Memorial Institute (RPMI) 1640 medium, fetal bovine serum (FBS), antibiotic-antimycotic solution 100X, Trypsin-EDTA Solution 1X, dimethyl sulphoxide (DMSO), Dulbecco’s phosphate buffered saline (DPBS) 1X and 3-(4, 5-dimethyle-2-thiazolyl)-2 and 5-diphenyl-2H-tetrazolium bromide (MTT) for biocompatibility studies were procured from HiMedia Laboratories Pvt. Ltd. Tissue culture treated plate (96-wells) and T-20 flask received from Costar 3599 and Nucleon respectively. MTT stock solution (5 mg/mL) was prepared in DPBS by dissolving MTT with the help of vortex. All chemicals were used as received without further purification.

### L-Cyst-Fe_3_O_4_ nanoparticle synthesis

L-Cyst functionalized magnetite nanoparticles (L-Cyst-Fe_3_O_4_ NPs) were synthesized using co-precipitation method discussed elsewhere^21^. Briefly, 16.2 g FeCl_3_ and 6.3 g FeCl_2_ (molar ratio 2:1) were dissolved and stirred in 100 mL distilled water for 1 h at 40 °C. A 2 M NaOH solution was added drop wise (1drop/2 sec) to Fe^2+^-Fe^3+^ solution for pH rise. During iron-oxide NPs formation, the suspension turned to black. Suspension was ultrasonicated for 30 min. A 0.2 M L-Cyst solution was prepared by adding 1.21 g of L-Cyst to 50 mL distilled water. The L-Cyst solution was added dropwise to NPs suspension and stirred for 20 min. Finally, NPs were separated by centrifugation (at 5500 rpm for 15 min).﻿ The separated NPs were washed for atleast five ti﻿mes with 25 mL distilled water and ethanol. NPs were dried overnight at 60 °C. The reaction mechanism for the formation of L-Cyst-Fe_3_O_4_ NPs is shown in Fig. S1 (Supplementary Information).

### Biocompatibility Studies

A549 human lung epithelial cancerous cells were incubated in RPMI 1640 medium with 10% FBS and 1% antibiotic-antimycotic solution 100X in 5% CO_2_ at 37 °C. Once cells achieved 90% confluency in T-20 culture flask then moved forward for MTT assay in 96-wells tissue culture treated plate. Initially, 5 × 10^3^ cells/well with 10% (FBS) RPMI-1640 were plated in 96-well tissue culture plate and incubated for next 24 h in 5% CO_2_ incubator at 37 °C. After achieving of 90% confluency, the media was replaced with 1% RPMI-1640 containing Fe_3_O_4_ and L-Cyst-Fe_3_O_4_ at different concentration ranges from 10 to 100 µg/mL and again incubated for next 24 h. Then 30 µL of MTT (5 mg/mL in DPBS) were added to each well. The tissue culture plate was kept in dark condition for 4 h in 5% CO_2_ incubator at 37 °C to allow the reduction of MTT dye to formazan crystal by living cells. After 4 h, whole media was removed and 200 µL of DMSO added to solubilize crystal. Absorbance was measured at 570 nm with the help of ELISA plate reader. The percentage (%) change in proliferation was calculated on control cells that were not exposed to NPs (i.e., only cells). All experiments were done in triplicate sets.

### Adsorption experiments

Adsorption studies for Pb^2+^ and Cr^6+^ removal by L-Cyst-Fe_3_O_4_ NPs were conducted in batch mode. Effect of pH on Pb^2+^ and Cr^6+^ was investigated at various initial pHs (pH 2.0 to 7.0 for Pb^2+^) and (pH 2.0 to 8.0 for Cr^6+^). Solutions pHs were adjusted using 1 M NaOH and 1 M HCl. The effect of contact time and adsorbent dose on Pb^2+^ and Cr^6+^ sorption were performed at different time intervals [5, 10, 15, 20, 25 and 35 minutes] and L-Cyst-Fe_3_O_4_ NPs dosage (1, 1.5, 2.0, 2.5 and 3.0 g/L). Sorption equilibrium studies were conducted by dispersing 0.1 mg adsorbent with 50 mL Pb^2+^ or Cr^6+^ solution [initial conc. = 10, 20, 40, 50, 60, 80, 100, 120, 150 mg/L; agitation speed = 200 rpm] at 25, 35 and 45 °C for 1 h. Upon reaching equilibrium, samples were filtered using a Whatman no. 42 filter paper. Equilibrium Pb^2+^ and Cr^6+^ concentrations were measured on AAS. The amount of Pb^2+^ and Cr^6+^ adsorbed on L-Cyst-Fe_3_O_4_ NPs per unit mass was calculated using eq. 1.

1 $${{\rm{q}}}_{{\rm{e}}}=\frac{({{\rm{C}}}_{{\rm{i}}}-{{\rm{C}}}_{{\rm{e}}})\ast {\rm{V}}}{{\rm{W}}}$$

Where, q_e_ is the amount of adsorbate adsorbed on per gram of adsorbent (mg/g), C_i_ & C_e_ are the initial and equilibrium concentrations (mg/L) of Pb^2+^ and Cr^6+^ in solution, V is the volume (L), and W is the weight (g) of the adsorbent.

### L-Cyst-Fe_3_O_4_ NPs characterization

The crytallinity and morphology of L-Cyst-Fe_3_O_4_ NPs were investigated on an X-ray powder diffractometer (model D/max 200, Rigaku) using a Cu-Kα radiation (λ = 1.54 Å; accelerating voltage = 40 KV; 2θ range = 10° to 80°; count rate = 1°/min). TEM micrographs were recorded using a transmission electron microscope (model JEM-2200 FS, JEOL) at an operating voltage of 200 KV. A pinch of sample was ultrasonicated with acetone for 10 min. An amorphous carbon coated copper grid was dipped in ultrasonicated solution and dried under vacuum. The grid was placed in the sample compartment to record the micrographs. The morphology, chemistry and elemental distribution of bare and loaded nanoparticle samples were carried out on a scanning electron microscope (model JSM-6480, JEOL; accelerating voltage = 20 KV). SEM elemental mapping images were captured using an EDS system (model Quantax 200, Bruker) supported with Esprit 1.8 software. A trace of sample was spread on a double stick carbon tape supported on an aluminum stub. Samples were made conductive by applying a gold coating using a sputter coater.

The optical studies of Fe_3_O_4_ and L-Cyst-Fe_3_O_4_ NPs were performed using a UV-Vis spectrophotometer (model Systronis-117) (λ range = 200–600 nm). A trace of sample was dispersed in 10 mL distilled water. Functional groups on bare and loaded adsorbents were identified using a Fourier transforms infrared spectrometer (FTIR; Model 600 UMA, Varian). Pellets were prepared by placing the NPs and KBr mixture (1:20 ratio) on a steel dye at a pressure of 10 tons using a hydraulic press.

The hydrodynamic radius of Fe_3_O_4_ and L-Cyst-Fe_3_O_4_ NPs was obtained from dynamic light scattering (DLS) measurements performed at a scattering angle, θ = 90° and laser wavelength of He/Ne laser (λ = 632.8 nm) using LS Spectrometer (model HNL210L, Thorlabs)^31^. The sample for analysis was prepared by dispersing pinch of NPs in 15 mL of distilled water. The hydrodynamic radius was obtained from Stoke-Einstein relation (eq. 2).

2 $${{\rm{R}}}_{{\rm{h}}}=\frac{{{\rm{k}}}_{{\rm{B}}}{\rm{T}}}{6{\rm{\pi }}{\rm{\eta }}{\rm{D}}}$$

Where, η is the solvent viscosity is, k_B_ is the Boltzmann constant, and T is the absolute temperature.

The point zero charge (pHpzc) of L-Cyst-Fe_3_O_4_ NPs were obtained from zeta-potential measurements performed on an electrophoresis instrument (model ZC-2000, Microtec, Japan). The samples were prepared by dispersing trace amount of NPs in 20 mL of distilled water followed by agitation for 48 h^32^. The pHpzc value was determined from a plot of initial pH versus pH of the supernatant.

Brunauer-Emmett-Teller (BET) surface area and porosity of synthesized Fe_3_O_4_ and L-Cyst-Fe_3_O_4_ NPs were determined by N_2_ adsorption-desorption studies using Quantachrome analyzer (model Autosorb-1). Samples (0.15 g) were degassed at 150 °C for 6 h at <10^−3^ Torr before adsorption measurements. Initial and equilibrium lead and chromium concentrations were measured using an atomic absorption spectrometer (AAS) (Model Aanalyst400, Perkin Elmer).

## Results and Discussion

### Characterization of L-Cyst-Fe_3_O_4_ NPs

#### X-ray powder diffraction (XRD)

XRD patterns of synthesized Fe_3_O_4_ and L-Cyst-Fe_3_O_4_ NPs are shown in Fig. S2 (Supplementary Information). The XRD patterns of Fe_3_O_4_ and L-Cyst-Fe_3_O_4_ NPs show diffraction miller planes at (200), (311), (400), (422), (511), (440) and (533) corresponding to the angle 2θ = 30°.091′, 35°.06′, 43°.076′, 53°.439′, 56°.96′, 62°.55′ and 74°.003′, respectively. The XRD peaks correspond to magnetite (JCPDS file no. 87–0245) and an inverse spinel and face centered cubic (fcc) structure. The XRD patterns confirm that L-Cyst functionalization on magnetite surface did not alter the phase and the nature of magnetite. Thus, L-Cyst-Fe_3_O_4_ is validated as Fe_3_O_4_. The average crystalline sizes of Fe_3_O_4_ and L-Cyst-Fe_3_O_4_ NPs were calculated using Debye-Scherer formula and most intense peak corresponding to (311) [eq. 3]. The average crystal sizes for Fe_3_O_4_ and L-Cyst-Fe_3_O_4_ NPs are 15 nm and 10.4 nm, respectively.

3 $${\rm{D}}=\frac{0.9{\rm{\lambda }}}{{\beta }\,\cos \,{\rm{\theta }}}$$

Where, 0.9 is the value assigned for constant ‘K’, which is related to the Miller index of crystallographic planes, λ is the wavelength of X-ray, θ is half of the diffraction angle and β is the angular width (in radians) at half-maximum intensity.

#### Transmission electron microscopy (TEM)

**Figure 1:**
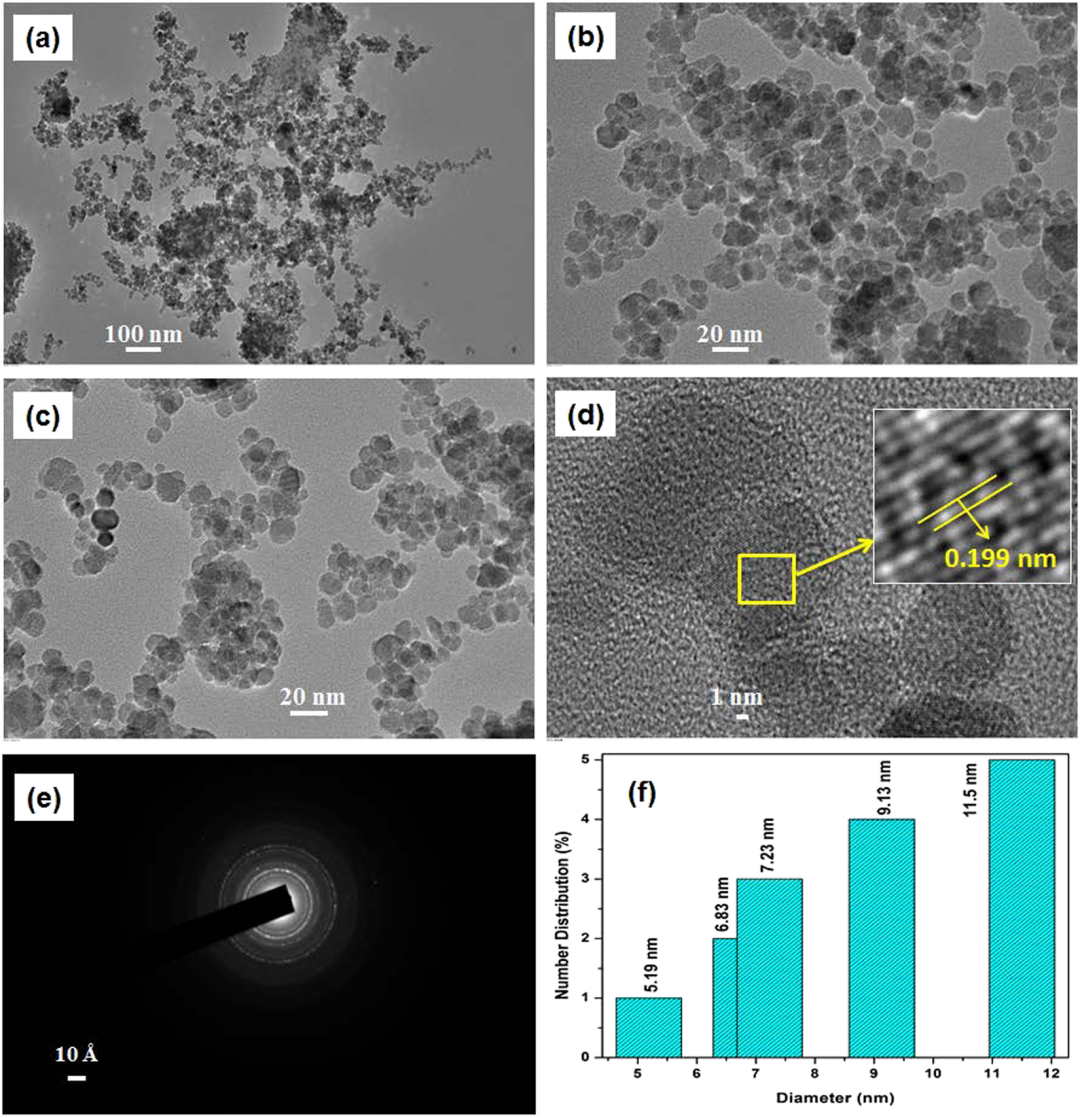
TEM images of L-Cyst-Fe_3_O_4_ NPs (**a**) at 20 kx; (**b**,**c**) at 10 kx magnification; (**d**) HR-TEM and inset shows lattice plane; (**e**) SAED pattern and (**f**) particle distribution of L-Cyst-Fe_3_O_4_ NPs.

Figure 1(a–c) shows TEM micrographs of L-Cyst-Fe_3_O_4_ NPs. The particles were highly dispersed as compared with bare Fe_3_O_4_ NPs [Fig. S3(a); (Supplementary Information)] and exhibited a uniform spherical morphology with an average crystal size of 10 nm. HRTEM captured lattice fringes (d = 0.199 nm) and confirmed a crystalline nature of NPs [image (d)]. The SAED patterns show the crytallinity of L-Cyst-Fe_3_O_4_ NPs [image (e)]. The particle size distribution of L-Cyst-Fe_3_O_4_ NPs ranges from 5 to 13 nm [image (f)].

#### Dynamic light scattering (DLS)

The hydrodynamic diameter provides information about inorganic core along with functionalized/coated material and the solvent layer attached to the particle as it moves under the influence of Brownian motion. The hydrodynamic sizes of Fe_3_O_4_ and L-Cyst-Fe_3_O_4_ NPs were 52 and 68 nm, respectively [Figure omitted for brevity]. The increase in the hydrodynamic size of functionalized magnetite NPs is due to an increase in adsorption of L-Cyst moieties onto the surface of the magnetite NPs^33^. This demonstrates a more hydrophilic nature of L-Cyst-Fe_3_O_4_ NPs than Fe_3_O_4_ NPs. Similar observations were reported earlier^27^. The size obtained from DLS was large as compared with TEM because in DLS dispersed NPs moves through a liquid medium and thin layer of electric dipole of the solvent adheres to its surface^31^.

#### Scanning Electron Microscopy (SEM)

The surface morphology of Fe_3_O_4_ and L-Cyst-Fe_3_O_4_ NPs were confirmed by SEM [Fig. S4(a–d); Supplementary Information]. The magnetite NPs were rough, globular and aggregated [Fig. S4(a,b)]. Impregnation of L-Cyst on Fe_3_O_4_ NPs is clearly visible in Fig. S4(c,d) (Supplementary Information). The morphological characteristics of L-Cyst-Fe_3_O_4_ NPs are favorable for metal adsorption. EDX analysis of Fe_3_O_4_ NPs shows the presence of iron (80.29%) and oxygen (19.71%) [Fig. S5(a); Supplementary Information]. Further, L-Cyst-Fe_3_O_4_ NPs show the presence of iron (90.93%), oxygen (5.23%), sulfur (2.17%), carbon (0.53%), and nitrogen (1.15%) [Fig. S5(b); Supplementary Information].

### Optical Studies

The optical absorption spectra of Fe_3_O_4_ and L-Cyst-Fe_3_O_4_ NPs are shown in Fig. S6(a) [Supplementary Information]. Absorption peak due to π-π* interaction appeared at 210 nm. Two absorption peaks at 300–365 nm (broad) and at 224 nm (sharp) represent the characteristic of iron NPs [Fig. S6(a)]. A sharp peak at 224 nm is due to the blue shift absorption band, thereby indicating the formation of polydisperse iron NPs^34^

**Figure 2:**
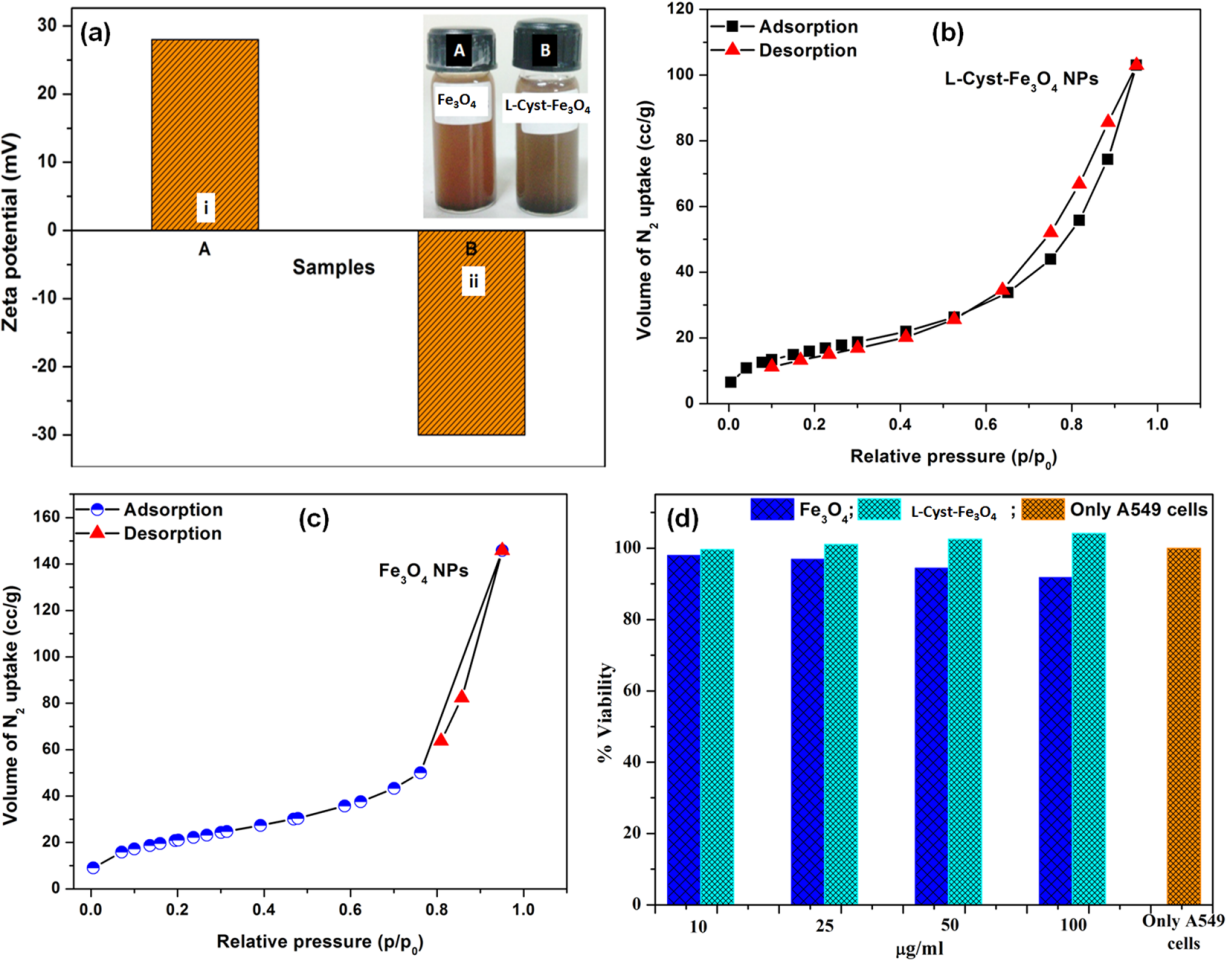
(**a**) Zeta potential of (i) Fe_3_O_4_ and (ii) L-Cyst-Fe_3_O_4_ NPs; (**b**) N_2_ adsorption-desorption isotherms of L-Cyst-Fe_3_O_4_ and (**c**) bare Fe_3_O_4_ NPs; (**d**) Comparative biocompatibility analysis of Fe_3_O_4_ and L-Cyst-Fe_3_O_4_ on A459 Cell line.

. The broad peak at 300–365 nm is due to Fe_3_O_4_ NPs local oxygen vacancies present in the lattice, indicating iron NPs formation^34, 35^. Wide peak was observed in the range 470–500 nm, which is attributed due to the formation of pair excitation of magnetically coupled Fe^3+^ ions^36, 37^. There was a decrease in the peak intensities at 228 and 300–365 nm after L-Cyst functionalization of magnetite. The peaks became wide. This indicated a decrease in electronic excitation state due to the formation of Fe-S bond.

The optical band gap energy was determined using the Tauc’s relation (eq. 4).

4 $$\begin{array}{ccc}{({\rm{\alpha }}h{\mathbb{V}})}^{\frac{1}{{\rm{n}}}} & = & {\rm{C}}(h{\mathbb{V}}-{{\rm{E}}}_{{\rm{g}}})\end{array}$$

Where, α is the absorption coefficient of NPs at a certain wavelength λ, h is Planck’s constant, C is a proportionality constant, Ѵ is the incident light frequency, E_g_ is the band gap energy, and the exponent, n = 1/2 and 2 for direct and indirect band gaps of NPs, respectively.

The band gaps of Fe_3_O_4_ and L-Cyst-Fe_3_O_4_ NPs were obtained by plotting curves between (*αh*Ѵ)^2^ and hѴ [Fig. S6(b); Supplementary Information]. The energy band gap (E_g_) of Fe_3_O_4_ (2.12 eV) was higher than those of bulk iron (2.0 eV)^35^. The band gap further decreased (1.4 eV) after L-Cyst impregnation. This could be possibly due to the hydrophilic nature of L-Cyst that resulted in peak broadening. Band gap widening is because of the striking quantum confinement effect observed in many other semiconducting mate rials that possess delocalized electronic states close to the Fermi level^38^. This confirms that L-Cyst is successfully funtionalized onto magnetite surface.

### Zeta Potential Study

The surface charges on synthesized Fe_3_O_4_ and L-Cyst-Fe_3_O_4_ NPs at pH 7 are shown in Fig. 2(a). The surface charge of magnetite in water could be explained by surface hydroxyl groups (Fe-OH). The Fe_3_O_4_ NPs zeta potential is positive (+28 mV), which are expected due to the formation of +Fe–OH_2_ in a basic environment [Fig. 2(a); sample (i)]. However, L-Cyst-Fe_3_O_4_ NPs have a negative zeta potential (−30.2 mV) [Fig. 2(a); sample (ii)]. The expected negative surface charge is due to the presence of carboxyl group above its isoelectric point [5.07]^27^. L-Cyst, like other amino acids, is a zwitterionic molecule^25, 27^. Thus, zeta potential of L-Cyst varies with pH^25, 30^. L-Cyst is negatively charged due to the presence of carboxyl group [pH < pH_pzc_ (5.07)] and positively charged due to the presence of ammonium group [pH > pH_pzc_ (5.07)]^27^. Thus, at pH 6.0, L-Cyst-Fe_3_O_4_ NPs exhibit a negative zeta potential^39^. This again confirmed the successful loading of L-Cyst on magnetite surface.

### Brunauer-Emmett-Teller (BET) Surface Area and Porosity Measurements

BET surface area and pore size distribution of bare Fe_3_O_4_ and L-Cyst-Fe_3_O_4_ NPs were determined by plotting the N_2_ adsorption-desorption isotherms [Fig. 2(b,c)]. N_2_ adsorption studies were performed by degassing the sample in vacuum at 150 °C for about 6 h. It is evident from Fig. 2(b,c) that N_2_ adsorption-desorption curve is a typical type IV adsorption isotherm with a hysteresis loop of type H_2_. Such type of isotherm explains the monolayer formation of adsorption^40^. The BET specific surface area of bare Fe_3_O_4_ and L-Cyst-Fe_3_O_4_ NPs, calculated from the nitrogen adsorption analysis were 75.69 and 58.49 m^2^/g, respectively. The average pore width was determined from distribution (PSD) curve using the Barrett-Joyner-Halenda (BJH) method and obtained as 11.9 and 10.8 nm for Fe_3_O_4_ and L-Cyst-Fe_3_O_4_ NPs, respectively. The pore size of Fe_3_O_4_ NPs varies from 16.8 to 11.9 nm and 6.2 to 10.8 nm for L-Cyst-Fe_3_O_4_ NPs, indicating that both the NPs were mesoporous in nature^41^. The total pore volume was obtained as 0.22 for Fe_3_O_4_ and 0.159 cm^3^/g for L-Cyst-Fe_3_O_4_ NPs. It is clear from comparative Table S1 (Supplementary Information), there is not much decrease in surface area and pore size of L-Cyst-Fe_3_O_4_ NPs versus Fe_3_O_4_ NPs, which is probably due to disruption in aggregation after surface modification^42^. In addition, this decrease in surface area possibly due to the presence of skinny layer of L-Cyst onto Fe_3_O_4_ NPs surface^42, 43^. Figure 2(b,c) shows BET constant [calculated from slope and intercept of the adsorption isotherm on the ordinate], significantly decreases after surface modification of Fe_3_O_4_ NPs. The BET theory signifies constant “C” as enthalpy of adsorption^42, 44^. Thus, its decrease indicates the decrease in interaction between adsorbate N_2_ molecules and magnetite surface after functionalized with L-Cyst. Similar results were earlier reported in literature^42–44^.

### Biocompatibility Studies

It was observed that the A549 cells viability increased after functionalization of L-Cyst onto Fe_3_O_4_ NPs. The L-Cyst-Fe_3_O_4_ NPs at the concentrations of 100, 50, 25, 10 µg/mL promoting cell proliferation by 104.15%, 102.56%, 101.09% and 99.63% versus bare Fe_3_O_4_ NPs at the concentrations of 100, 50, 25, 10 µg/mL [Fig. 2(d)]. This may be due to Cysteine moiety.

### Pb^2+^ and Cr^6+^ adsorption on L-Cyst-Fe_3_O_4_ NPs

#### Effect of Initial pH

**Figure 3:**
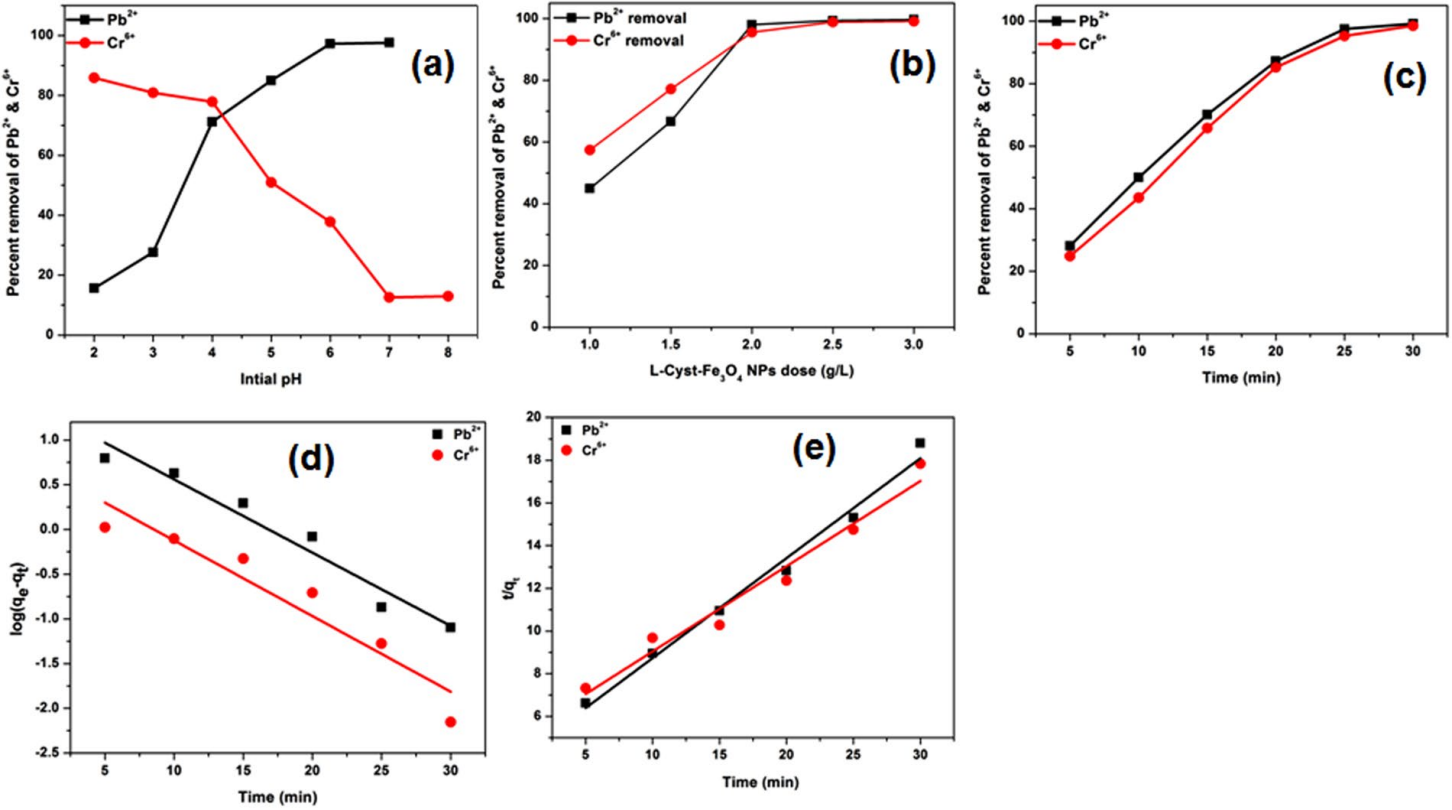
Pb^2+^ and Cr^6+^ curves obtained at different (**a**) initial pH; (**b**) L-Cyst-Fe_3_O_4_ NPs dose; (**c**) Effect of contact time; (**d**) Pseudo-first-order and (**e**) pseudo-second-order kinetic plots.

The solution pH provides information about effect of adsorbent functional groups, degree of ionization of adsorbate, the metal ions solubility, and the concentration of counter ions during reaction^40^. Adsorption studies were carried out in the pH range of 2.0–7.0 for Pb^2+^ and 2.0–8.0 for Cr^6+^ at 25 °C (equilibrium time: 60 min; agitation speed: 200 rpm; adsorbent dose: 2.0 g/L; adsorbate concentration: 50 mg/L). pH adjustments were made using 1 M NaOH and 1 M HCl. Pb^2+^ removal was increased with increase in pH [Fig. 3(a)]. At pH 6.0, lead exists as Pb^2+^ and L-Cyst-Fe_3_O_4_ NPs surfaces possess a negative charge [zeta potential: −14.69 mV] as shown in Fig. S7 (Supplementary Information). Thus, adsorbent-adsorbate electrostatic interactions take place^45^. L-Cyst-Fe_3_O_4_ NPs exhibit high removal at pH > 2. With pH rise, carboxylate group density increase at NPs interface and in the dispersion medium^25, 27, 28^. Pb^2+^ adsorption is less at low pH [Fig. S7 (Supplementary Information)]. This is because in acidic medium, the carboxylate and amine groups on L-Cyst gets protonated and act as cation [Fig. S7 (Supplementary Information)]. As a result, L-Cyst-Fe_3_O_4_ surface has a net positive charge and there is repulsion of ions. This lowers the overall adsorption capacity. Pb (OH) _2_ precipitation starts after pH 6.0﻿^40, 46^.

**Figure 4:**
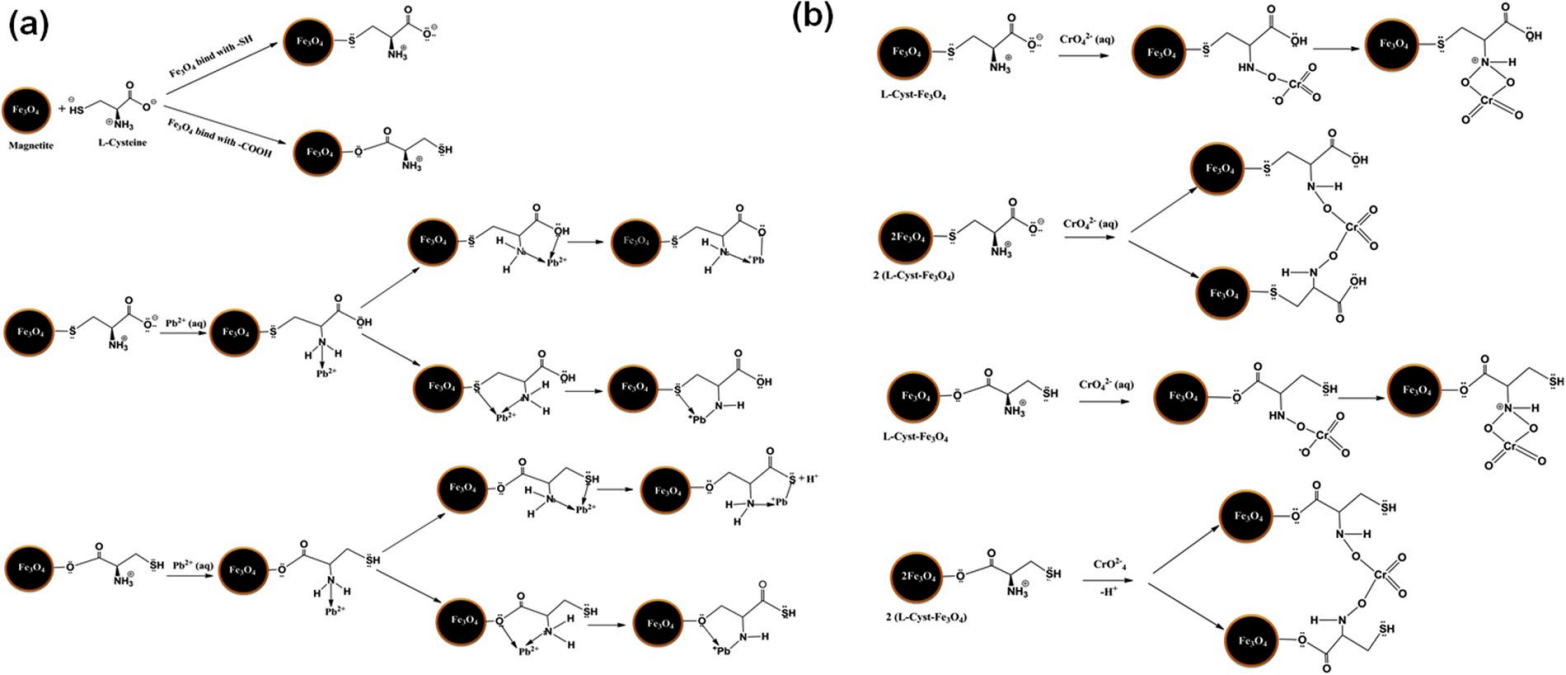
The binding mechanism of (**a**) Pb^2+^ and (**b**) Cr^6+^ ions onto L-Cyst-Fe_3_O_4_ NPs.

The Cr^6+^ removal efficiency of L-Cyst-Fe_3_O_4_ NPs decreased after pH > 3.0 [Fig. 3(a)]. Therefore, pH 3.0 was considered as an optimum pH for further Cr^6+^ adsorption studies. Chromium exists as HCrO_4_^−^ and Cr_2_O_7_^2−^ and L-Cyst-Fe_3_O_4_ NPs are positively charged at an aqueous pH < 3.0^27, 40, 46^ [Fig. S7 (Supplementary Information)]. Therefore, there is an electrostatic attraction between negatively charged ions (HCrO_4_^−^ and Cr_2_O_7_^2−^) and L-Cyst-Fe_3_O_4_ NPs (L-Cyst-Fe_3_O_4_ pHpzc = 5.7). However at pH > 3, removal efficiency decreases. At pH > 3.0, repulsion between L-Cyst-Fe_3_﻿O_4_ NPs and chromate ions takes place which results into decrease in adsorption capacity [Fig. 3(a)]. The possible binding mechanisms of Pb^2+^ and Cr^6+^ onto L-Cyst-Fe_3_O_4_ NPs are shown in Fig. 4(a,b).

#### Effect of L-Cyst-Fe_3_O_4_ NPs Dose

The effect of L-Cyst-Fe_3_O_4_ NPs dose on Pb^2+^ and Cr^6+^ uptake is shown in Fig. 3(b). Pb^2+^ and Cr^6+^ sorption experiments were conducted using 50 mL Pb^2+^ or Cr^6+^ solution (50 mg/L) and L-Cyst-Fe_3_O_4_ NPs (dosage: 1.0, 1.5, 2.0, 2.5 and 3.0 g/L) at optimal conditions [temperature: 25 °C, agitation speed: 200 rpm, equilibrium time: 60 min]. Pb^2+^ and Cr^6+^ removal increased considerably on increasing the dose (from 1.0 to 3.0 g/L) due to increase in the number of sorption sites^40, 46, 47^. Pb^2+^ removal increased from 45% to 99% on increasing dose from 1.0 to 2.0 g/L. No further Pb^2+^ uptake was observed on increasing adsorbent dose to 2.5 and 3.0 g/L. Similarly, Cr^6+^ uptake increased from 62% to 96% on increasing dose from 1.0 to 2.0 g/L. Therefore, 2.0 g/L dose was used as optimum dose for further Pb^2+^ and Cr^6+^ adsorption.

#### Effect of Contact Time

Pb^2+^ and Cr^6+^ adsorption experiments were conducted at optimal conditions [pH = 3.0 for Cr^6+^ and 3.0 for Pb^2+^; adsorbent dose: 2.0 g/L, adsorbate: 50 mg/L, temperature: 25 °C, agitation speed: 200 rpm, equilibrium time: 60 min] and different contact time (5, 10, 15, 20, 25, 30 min). Pb^2+^ and Cr^6+^ removal increased with increase in contact time (from 5 to 30 min) [Fig. 3(c)]. Almost complete Cr^6+^ and Pb^2+^ removal occurred in 25 min. No further uptake was recorded after 30 min. The removal rate was initially high and slowed down as the equilibrium approached.

#### Cr^6+^ and Pb^2+^ Sorption Dynamics

Cr^6+^ and Pb^2+^ adsorption kinetics were fitted to pseudo-first-order^47^ and pseudo-second-order^48, 49^ rate equations (eqs 5 and 6).

5 $${\rm{l}}{\rm{n}}\,({{\rm{q}}}_{{\rm{e}}}-{{\rm{q}}}_{{\rm{t}}})=\,{\rm{l}}{\rm{n}}({{\rm{q}}}_{{\rm{e}}})-{{\rm{k}}}_{1}{\rm{t}}$$

6 $$\frac{{\rm{t}}}{{{\rm{q}}}_{{\rm{t}}}}=\frac{1}{{{\rm{k}}}_{2}{{{\rm{q}}}_{{\rm{e}}}}^{2}}+\frac{{\rm{t}}}{{{\rm{q}}}_{{\rm{e}}}}$$

Here, k_1_ and k_2_ (min^−1^) are the first-order and second-order adsorption rate constants, q_e_ is the amount adsorbed at equilibrium, and q_t_ is the amount of Pb^2+^ and Cr^6+^ adsorbed at time ‘t’.

Figure 3(d,e) shows the pseudo-first and second-order kinetic plots obtained for Pb^2+^ and Cr^6+^ adsorption on L-Cyst-Fe_3_O_4_ NPs. The slopes and intercepts as calculated from the plots were used to determine the regression coefficient (R^2^) and kinetic rate constants (k_1_ and k_2_) [eqs 5 and 6]. The kinetic parameters obtained are summarized in Table 1. Pb^2+^ and Cr^6+^ adsorption data well fitted to pseudo-second order equation [R^2^ > 0.985 (Pb^2+^); 0.961 (Cr^6+^)]. Thus, chemisorption is the rate determining step for Cr^6+^ and Pb^2+^ adsorption on L-Cyst-Fe_3_O_4_ NPs. Similar observations were reported earlier ﻿for Pb^2+^ and Cr^6+^ ﻿adsorption ﻿on magnetite nanospheres and magnetite nanoparticles^40, 46^.

**Table 1:** Pseudo-first and second-order rate constants and correlation coefficients obtained for Cr^6+^ and Pb^2+^ adsorption on L-Cyst-Fe_3_O_4_ NPs.

Metals	Pseudo-first-order rate constants		Pseudo-second-order rate constants	
	k_1_	R^2^	k_2_	R^2^
Pb^2+^	0.08	0.9478	0.47	0.9850
Cr^6+^	0.08	0.8930	0.40	0.9611

#### Cr^6+^ and Pb^2+^ Sorption Equilibrium Studies

Sorption isotherm models provide information about the adsorption mechanisms and adsorbate-adsorbent interactions. Cr^6+^ and Pb^2+^ sorption equilibrium data were fitted to the Langmuir and Freundlich equations. The Langmuir isotherm assumes that the adsorption process takes place on monolayer adsorbent surfaces with uniform energy^50, 51^, while Freundlich isotherm assumes that the adsorption process occurs on multilayer surfaces with non-uniform distribution of energy^52, 53^. The linear form of Langmuir and Freundlich isotherms are given by eqs 7 and 8, respectively.

7 $$\frac{{{\rm{C}}}_{{\rm{e}}}}{{{\rm{q}}}_{{\rm{e}}}}=\frac{1}{{{\rm{bq}}}_{{\rm{\max }}}}+\frac{{{\rm{C}}}_{{\rm{e}}}}{{{\rm{q}}}_{{\rm{\max }}}}$$

8 $$\mathrm{ln}\,{{\rm{q}}}_{{\rm{e}}}=\,\mathrm{ln}\,{{\rm{K}}}_{{\rm{F}}}-\frac{1}{{\rm{n}}}\,\mathrm{ln}\,{{\rm{C}}}_{{\rm{e}}}$$

Where, b (L/mg) is the Langmuir adsorption constant and q_max_ (mg/g) is the monolayer adsorption capacity of the adsorbent. K_F_ and 1/n are Freundlich constants which correspond to adsorption capacity and adsorption intensity, respectively. All the Freundlich and Langmuir constants are given in Table 2. The Langmuir separation factor (R_L_) (eq. 9) can also be used to predict the affinity between the adsorbate and adsorbent.

9 $${{\rm{R}}}_{{\rm{L}}}=\frac{1}{(1+{{\rm{bC}}}_{{\rm{i}}})}$$

Where b is the Langmuir constant and C_i_ is the initial concentration. The value of R_L_ tells the type of isotherm to be irreversible (R_L_ = 0), linear (R_L_ = 1), unfavorable (R_L_ > 1), or favorable (0 < R_L_ < 1)^48^. R_L_ values between 0 and 1 indicate favorable Pb^2+^ and Cr^6+^ adsorption on L-Cyst Fe_3_O_4_ NPs [Fig. S8(a,b)] .

**Figure 5:**
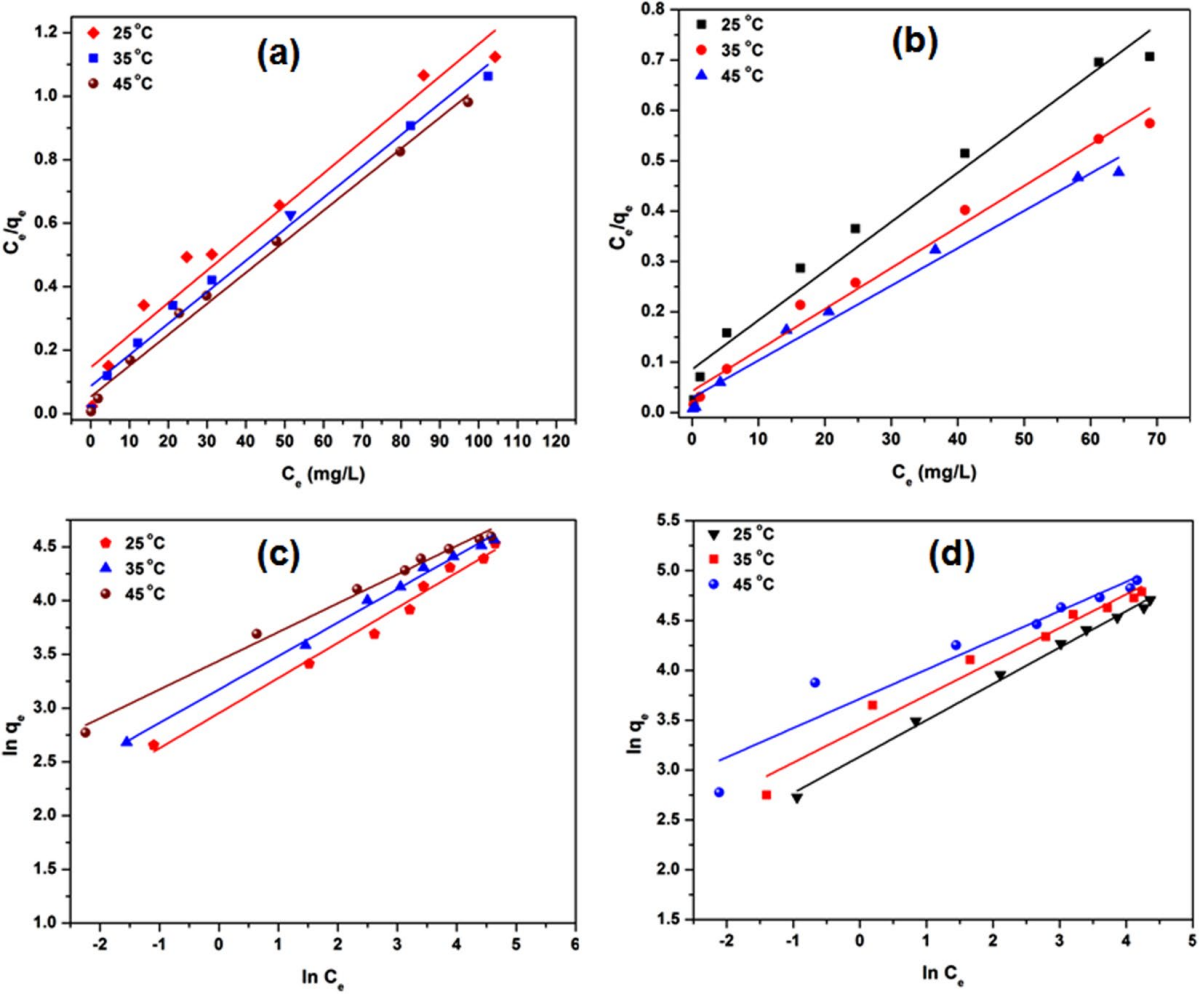
Langmuir isotherm plots for (**a**) Pb^2+^ and (**b**) Cr^6+^ adsorption; Freundlich isotherm plots for (**c**) Pb^2+^ and (**d**) Cr^6+^ adsorption.

The linear plots of both Langmuir and Freundlich isotherm model of Pb^2+^and Cr^6+^ adsorption are shown in Fig. 5(a–d). The Langmuir adsorption equilibrium constants of Pb^2+^ and Cr^6+^ were obtained from linear plots between C_e_/q_e_ and C_e_ [eq. 7] [Fig. 5(a,b)]. The Freundlich equilibrium constants were determined from the plot of ln q_e_ versus ln C_e_ [eq. 8] [Fig. 5(c,d)]. The Pb^2+^ and Cr^6+^ uptake increased with temperature rise from 25 °C to 45 °C indicating the endothermic nature of adsorption. The adsorption constants and correlation coefficients (R^2^) of Langmuir and Freundlich models are given in Table 2. The Langmuir equation best fitted the data obtained for Pb^2+^ and Cr^6+^ adsorption onto L-Cyst-Fe_3_O_4_ NPs [Table 2], thereby indicating a monolayer adsorption of Pb^2+^ and Cr^6+^. A maximum adsorption capacity (q_e_) of L-Cyst-Fe_3_O_4_ NPs was 18.78 mg/g (for Pb^2+^) and 34.48 mg/g (for Cr^6+^).

**Table 2:** Langmuir and Freundlich isotherm parameters obtained for Cr^6+^ and Pb^2+^ adsorption on L-Cyst-Fe_3_O_4_ NPs.

Isotherm constants	Pb^2+^			Cr^6+^		
	25 °C	35 °C	45 °C	25 °C	35 °C	45 °C
**Langmuir**						
q_max_ (mg/g)	7.00	11.60	18.80	11.66	23.48	34.48
b (L/mg)	14.26	8.72	5.45	8.79	5.23	4.14
R^2^	0.956	0.987	0.991	0.971	0.984	0.986
**Freundlich**						
K_F_ (mg/g)	19.16	23.88	31.19	25.98	30.27	41.05
n	3.07	3.22	3.74	1.44	3.03	3.41
R^2^	0.982	0.995	0.990	0.994	0.966	0.914

#### Adsorption Thermodynamics.

 The Gibbs free energy of adsorption [ΔG° (kJ/mol)], the enthalpy change [ΔH° (kJ/mol)], and the entropy change [ΔS° (kJ/(mol K))] were determined using eqs 10, 11, 12, and 13 ^54, 55^.﻿

10 $$\frac{{\rm{d}}{\rm{l}}{\rm{n}}{\rm{K}}}{{\rm{d}}{\rm{t}}}=\frac{{{\rm{\Delta }}{\rm{H}}}^{^\circ }}{{{\rm{R}}{\rm{T}}}^{2}}$$

Considering that ΔH does not change much with change in temperature over the temperature range of study, the integration of (eq. 10) results into:

11 $${\rm{l}}{\rm{n}}\,{\rm{K}}=-\frac{{{\rm{\Delta }}{\rm{H}}}^{^\circ }}{{\rm{R}}{\rm{T}}}+C$$

**Figure 6:**
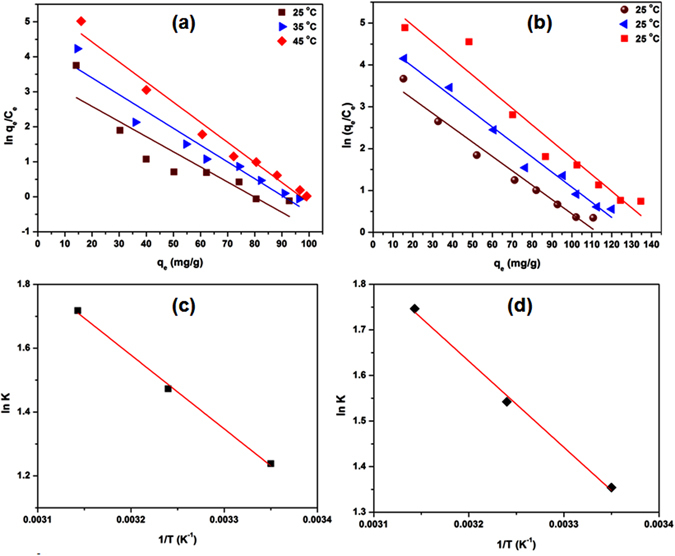
(**a**) ln (q_e_/C_e_) against q_e_ plots at different temperatures (25, 35 and 45 °C) for Pb^2+^; (**b**) Cr^6+^ adsorption and (**c**) Van’t Hoff plot for Pb^2+^; (**d**) Van’t Hoff plot for Cr^6+^ adsorption.

Where, K is the thermodynamic equilibrium constant, ΔH° is the standard molar sorption enthalpy at temperature T, R is the universal gas constant (8.314 J/mol K), C is integration constant and T is the absolute temperature (K). The values of K were determined by plotting ln (q_e_/C_e_) against q_e_ [Fig. 6(a,b)], where q_e_ is the amount of Pb^2+^ and Cr^6+^ adsorbed and C_e_ is the Pb^2+^ and Cr^6+^ equilibrium concentrations. The plot of ln K against 1/T [Fig. 6(c,d)] ﻿theoretically yields a straight line allowing ﻿calculation of ∆H° and ∆S° from the respective slope (equal to −ΔH°/R) and intercept (﻿equal to ΔS°/R﻿) of eq. 1﻿2.

12 $${\rm{l}}{\rm{n}}\,{\rm{K}}=-\frac{{{\rm{\Delta }}{\rm{H}}}^{\circ }}{{\rm{R}}{\rm{T}}}+\frac{{{\rm{\Delta }}{\rm{S}}}^{\circ }}{{\rm{R}}}$$

The value of standard Gibbs free energy (ΔG°) was calculated using eq. 13.

13 $${{\rm{\Delta }}{\rm{G}}}^{\circ }=-{\rm{R}}{\rm{T}}\,{\rm{l}}{\rm{n}}({{\rm{K}}}_{})$$

Where, R is the universal constant (8.314 J/mol K) and T is the absolute temperature (K).

The values of the thermodynamic parameters are given in Table 3. ΔH° and ΔS° were positive and ΔG° was negative in all systems. The positive values of ΔH° [83.7 KJ mol^−1^ (for Pb^2+^); 73.31 KJ mol^−1^ (for Cr^6+^)] indicated an endothermic nature of adsorption process^55^. Further, the positive ΔS° values [0.31 (for Pb^2+^); 0.28 (for Cr^6+^)] revealed the increased randomness at solid/solution interface^50, 55, 56^. The ΔG° values decreased with rise in temperature. The negative values of ΔG° [−8.55 (T = 25 °C), −11.17 (T = 35 °C), −14.7 (T = 45 °C) for Pb^2+^; −9.60 (T = 25 °C), −11.98 (T = 35 °C), −15.16 (T = 45 °C) for Cr^6+^] indicated that sorption was spontaneous and thermodynamically favorable.

**Table 3:** Thermodynamic parameters for Pb^2+^ and Cr^6+^ adsorption onto L-Cyst-Fe_3_O_4_ NPs.

Adsorbate	ΔH° (kJ/mol)	ΔS° [kJ/(mol K)]	T (K)	K (L g^−1^)	ΔG° (kJ/mol)
Pb^2+^	83.7	0.31	298.15	3.448	−8.55
			308.15	4.360	−11.17
			318.15	5.571	−14.7
Cr^6+^	73.31	0.28	298.15	3.873	−9.60
			308.15	4.675	−11.98
			318.15	5.733	−15.16

#### Characterization of Pb^2+^ and Cr^6+^ loaded L-Cyst-Fe_3_O_4_ NPs

 Pb^2+^ and Cr^6+^ uptake onto L-Cyst-Fe_3_O_4_ NPs was further confirmed by carrying out FT-IR, SEM-EDX and SEM mapping studies of Pb^2+^ and Cr^6+^ loaded L-Cyst-Fe_3_O_4_ NPs. About 0.5 g/L of L-Cyst-Fe_3_O_4_ NPs were agitated with in 50 mg/L Pb^2+^ (pH 5.0) and Cr^6+^ (pH 3.0) solution at optimal conditions (temperature: 25 °C, agitation speed: 200 rpm; equilibrium time: 60 min). The suspension was filtered using Whatman filter paper no. 42 and the residue was dried in an oven for 50 °C over night.

#### FTIR studies

**Figure 7:**
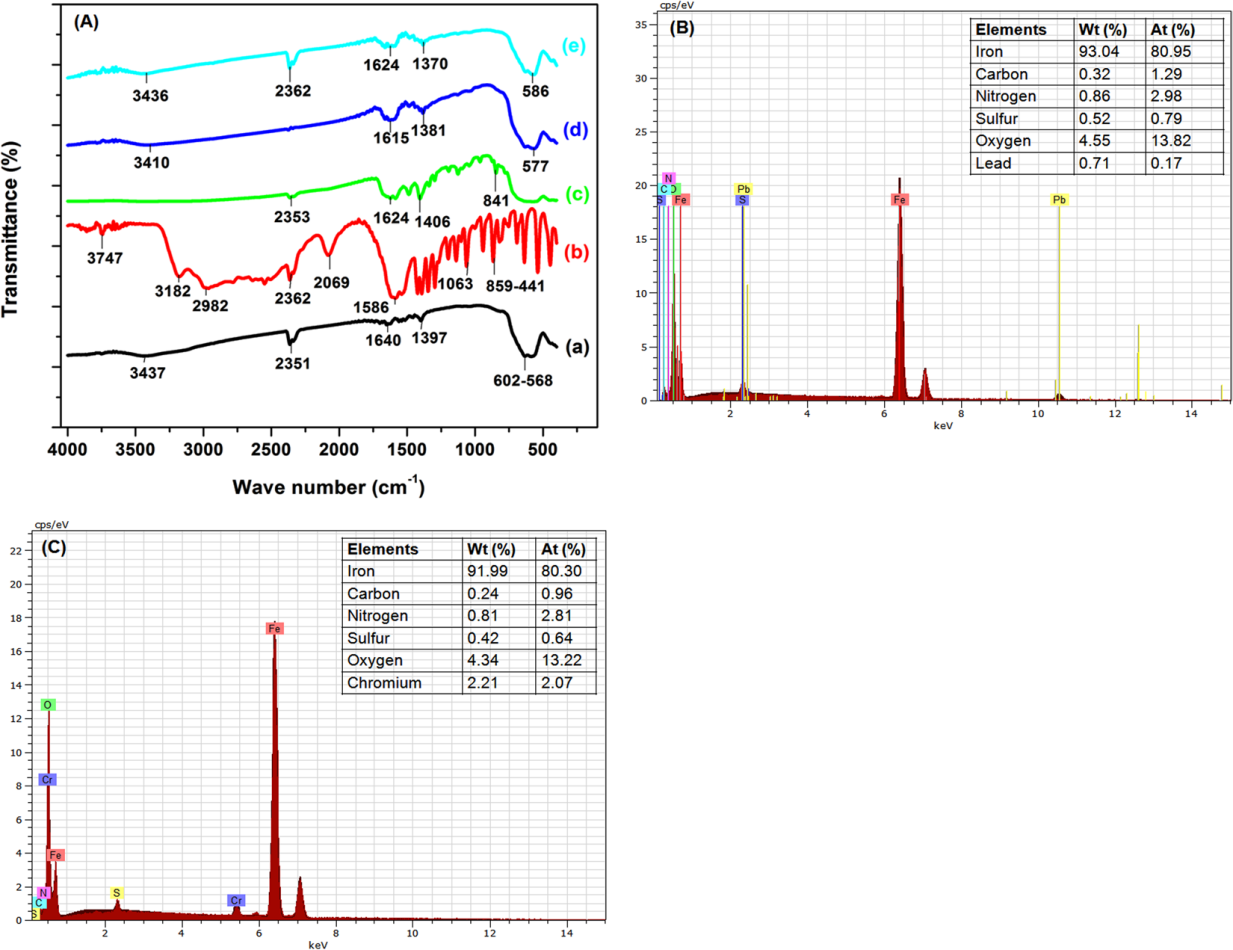
(**A**) FTIR spectra of (a) Fe_3_O_4_, (b) commercial L-Cyst, (c) L-Cyst-Fe_3_O_4_, (d) Pb^2+^ loaded L-Cyst-Fe_3_O_4_ and (e) Cr^6+^ loaded L-Cyst-Fe_3_O_4_; (**B**) EDX spectra of Pb^2+^ loaded L-Cyst-Fe_3_O_4_ NPs and (**C**) Cr^6+^ loaded L-Cyst-Fe_3_O_4_ NPs.

The FTIR spectra of bare Fe_3_O_4_, commercial L-Cyst, L-Cyst-Fe_3_O_4_ NPs, Pb^2+^ and Cr^6+^ loaded L-Cyst-Fe_3_O_4_ NPs were obtained in the range of 400–4000 cm^−1^ [Fig. 7A(a–e)]. The sharp band of magnetite appeared at 568 cm^−1^ and became less intense at 602 cm^−1^ ^57^. A strong band at 568 cm^−1^ is a characteristic of Fe-O absorption due to its symmetric stretching vibration^27^. Sharp peaks at 1640 and 3437 cm^−1^ were attributed to O-H stretching and bending vibrational bands of water moiety adsorbed on magnetite surface [curve (a)]. A strong peak at 2351 cm^−1^ attributes to carbon dioxide (ѵ CO_2_)^55, 57, 58^. A curve (b) shows FT-IR spectrum of commercial L-Cyst. This curve exhibits its characteristic bands at 1602 and 1390 cm^−1^ due to the asymmetric and symmetric stretching of COO^−^^27^. The band at 1586 cm^−1^ corresponds to N–H bending. A sharp peak of NH_3_^+^ stretch is observed between 2982–3182 and 3747 cm^−1^. A weak band at 2550–2362 cm^−1^ is due to the presence of S–H group^59^. However, after the functionalization of L-Cyst onto the surface of magnetite NPs, some new peaks emerged at 1624, 1590 and 1406 cm^−1^ corresponding to asymmetric and symmetric stretching of COO^−^ and -NH_2_. Amide band indicates the successful functionalization of L-Cyst molecules onto magnetite surface. In addition, the sharp peak at 2069 cm^−1^ disappeared [curve (c)], implying the formation of the covalent bond between Fe and S. Similar observations were reported earlier for L-Cyst capped Fe_3_O_4_ NPs^21, 27^.

However, after Pb^2+^ and Cr^6+^ uptake on L-Cyst-Fe_3_O_4_ NPs, some shifting in the peaks between 1624 and 1401 cm^−1^ (corresponding to asymmetric and symmetric stretching of COO^−^ and -NH_2_, respectively) were observed. A sharp peak at 1509 disappeared [curve (d, e)]. This could be probably due to binding of Pb^2+^ and Cr^6+^ with COO^−^ and -NH_2_ groups. The peaks between 3410 to 3416 cm^−1^ weakens after Pb^2+^ and Cr^6+^ uptake. It may be due to surface precipitation of lead hydroxide and chromium hydroxide that decreased the intensity of O–H stretching frequencies^46^.

#### SEM-EDX and mapping

Figure 7(B,C) shows the SEM-EDX spectra of Pb^2+^ and Cr^6+^ loaded NPs. Inset Tables in Fig. 7(B,C) summarizes intense EDX peaks of lead and chromium, which clearly confirmed the adsorption of Pb^2+^ and Cr^6+^ on L-Cyst-Fe_3_O_4_ NPs surfaces.

**Figure 8:**
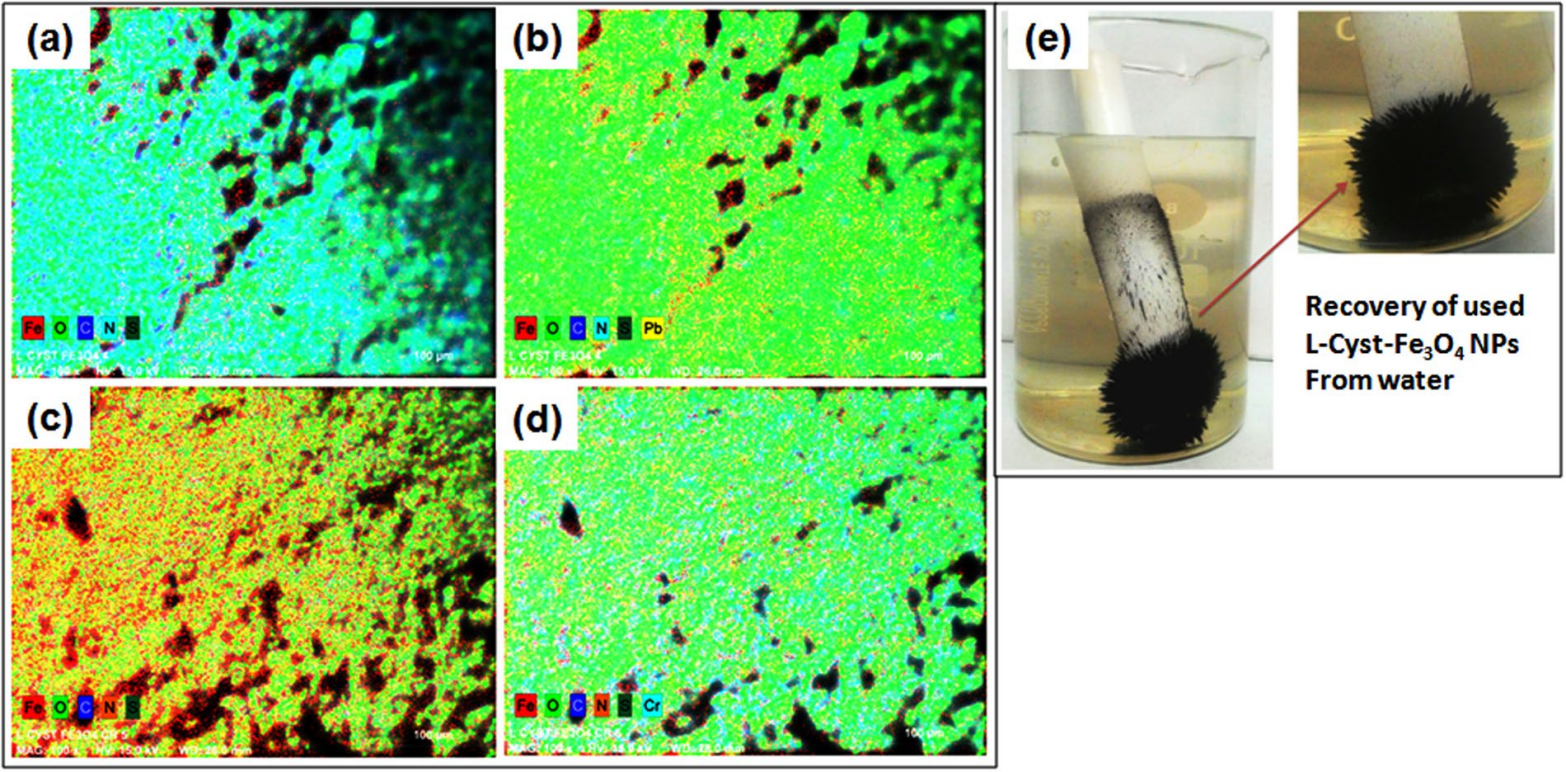
SEM elemental mapping images of (**a**,**c**) L-Cyst-Fe_3_O_4_ NPs; (**b**) Pb^2+^ loaded L-Cyst-Fe_3_O_4_ NPs (Lead in yellow); (**d**) Cr^6+^ loaded L-Cyst-Fe_3_O_4_ NPs (Chromium in cyan color) and (**e**) Demonstration of exhausted L-Cyst-Fe_3_O_4_ NPs separation from aqueous system.

SEM mapping micrographs of L-Cyst-Fe_3_O_4_ NPs before and after uptake of Pb^2+^ and Cr^6+^ are shown in Fig. 8(a–d). The elemental distribution specifies iron in red, oxygen in fluorescent green, carbon in blue, nitrogen in cyan and sulfur in dark greens [Fig. 8(a,c)]. The elemental mapping also confirmed the Pb^2+^ uptake (in yellow) [Fig. 8(b)] and Cr^6+^ (in cyan) [Fig. 8(d)]. It is clear, Pb^2+^ and Cr^6+^ ions are uniformly distributed on L-Cyst-Fe_3_O_4_ NPs surfaces [Fig. 8(b,d)].

Recovery of exhausted L-Cyst-Fe_3_O_4_ NPs from aqueous system using a simple magnet is demonstrated in Fig. 8(e). The NPs were readily attracted to the magnet. Therefore, the NPs can easily be recovered using a magnet rather traditional filtration.

Table S2 (Supplementary Information) shows the comparative adsorption capacities of L-Cyst-Fe_3_O_4_ NPs for Pb^2+^ and Cr^6+^ removal versus other nanosorbents^40, 60–68^. The L-Cyst-Fe_3_O_4_ NPs adsorption capacity is comparable to other similar adsorbents and can replace the costly commercial adsorbents.

#### Regeneration and Stability of L-Cyst-Fe_3_O_4_ NPs

**Figure 9:**
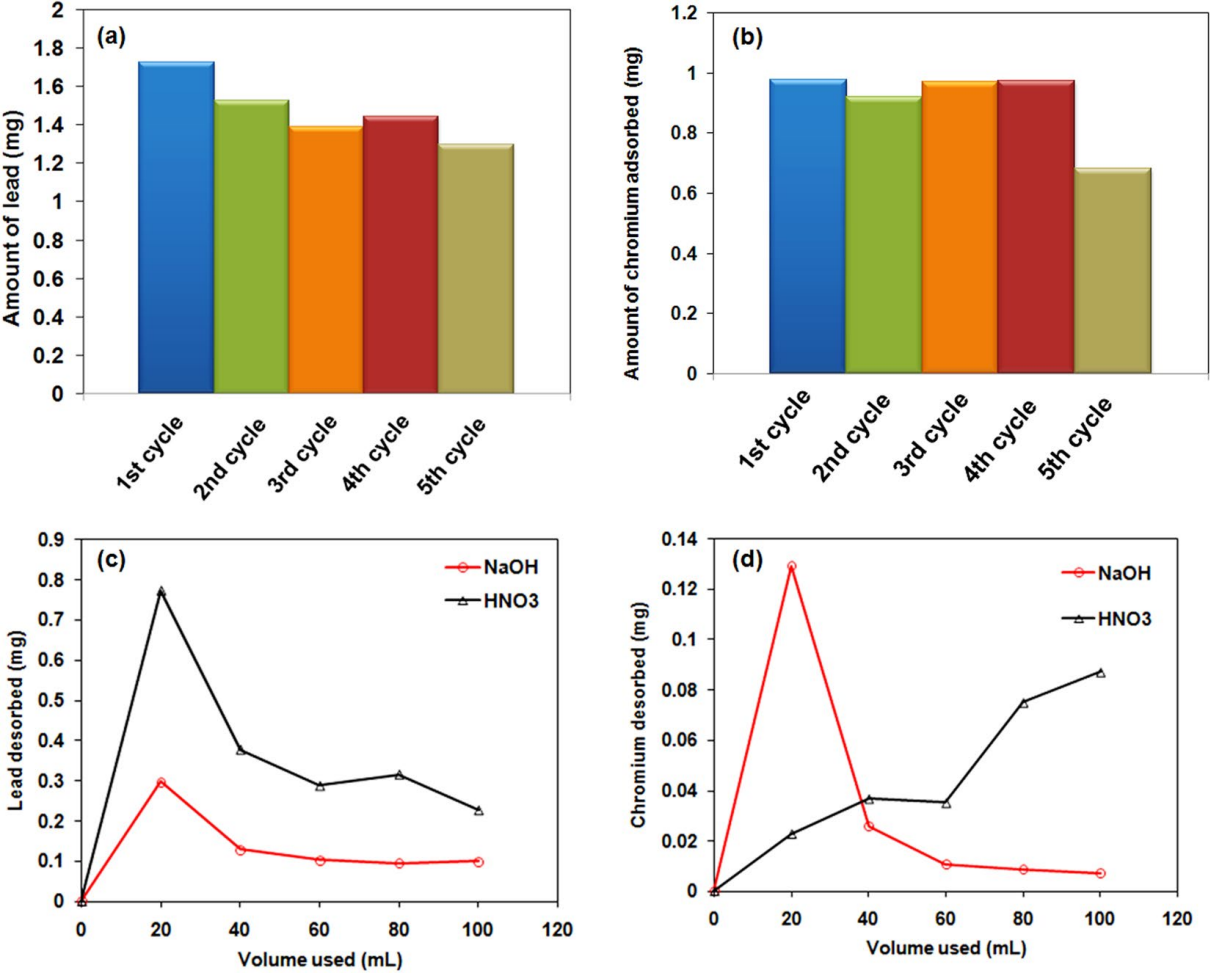
Adsorption cycles of (**a**) lead and (**b**) chromium of the L-Cyst-Fe_3_O_4_ NPs at 25 °C [adsorbate conc. = 50 mg/L; adsorbent conc. = 3.0 g/L]; desorption cycles of (**c**) lead and (**d**) chromium of the L-Cyst-Fe_3_O_4_ NPs at 25 °C [adsorbate conc. = 50 mg/L; adsorbent conc. = 3.0 g/L; desorption agent: 20 mL of 0.01 M HNO_3_ and NaOH].

The efficiency of NPs was investigated for multiple adsorptions-desorption cycles. Briefly, 3.0 g/L NPs were agitated with 50 mg/L Pb^2+^ or Cr^6+^ solution (40 mL) for 15 minutes. Equilibrium concentration was measured and the spent NPs were desorbed using 0.01 M NaOH or HNO_3_. The desorbed NPs were dried and reused for another adsorption cycle (Pb^2+^ and Cr^6+^ separately). This adsorption-desorption procedure was repeated five times [Fig. 9(a,b)]. The adsorption yield decreased in every cycle. Lead adsorption yield was 83% in first cycle that reduced to 37% in fifth cycle [Fig. 9(a)]. However, chromium removal yield was slight better. It was 22% in the first cycle that reduced to 15% in the fifth cycle [Fig. 9(b)].

 Almost 40% of the total desorption was achieved in first 20 mL HNO_3_ or NaOH for lead [Fig. 9(c)]. Lead desorption was favorable using an acidic eluent because at low pH, there is a competition between H^+^ ions and metal ions as demonstrated in Fig. 4(a) where the surface FeO^−^ react with H^+^ or Pb^2+^. A 46% and 71% of total chromium desorption was achieved using first 20 mL HNO_3_ and NaOH, respectively [Fig. 9(d)]. Thus, magnetite nanoparticles can be regenerated and reused for Cr^6+^ and Pb^2+^ removal and recovery.

Furthermore, TEM studies were also performed to check the stability and morphology of spent nanosorbents after five successive adsorption-desorption cycles. Figure S9(b,c) (Supplementary Information) shows the TEM images of regenerated L-Cyst-Fe_3_O_4_ NPs retained almost the original morphology after adsorption-desorption cycles. These results suggest that L-Cyst-Fe_3_O_4_ NPs has good reusability and stability properties.

## Conclusions

Highly stable L-Cyst functionalized magnetite NPs were successfully synthesized by co-precipitation. L-Cyst effectively prevents magnetite NPs from oxidation, aggregation and also increased its biocompatibility. SEM, energy band gap, zeta potential, and hydrodynamic size studies confirmed the successful impregnation of L-Cyst onto magnetite NPs surface. The absence of NH_3_^+^ vibrational peak and shifting of IR bands implied the successful L-Cyst functionalization onto magnetite surface as well as Pb^2+^ and Cr^6+^ loading. L-Cyst interacts with the NPs via its thiol group and heavy metals *via* its amino and carboxyl groups. SEM-EDX and SEM mapping images confirmed successful adsorption of Pb^2+^ and Cr^6+^ onto L-Cyst-Fe_3_O_4_ NPs. Sorption equilibrium was reached within 25 min at 25 °C. Langmuir equation best described the sorption equilibrium data. Langmuir adsorption capacities of 34.5 and 18.8 mg g^−1^ were obtained for Cr^6+^ and Pb^2+^, respectively. Sorption dynamics data were best described using pseudo-second-order rate equation. Maximum Pb^2+^ and Cr^6+^ removal was achieved at pH 6.0 and 2.0, respectively. Thermodynamic studies illustrated that Cr^6+^ and Pb^2+^ adsorption is endothermic in nature. The manipulation of exhausted L-Cyst-Fe_3_O_4_ NPs from aqueous media was also demonstrated. L-Cyst-Fe_3_O_4_ NPs stability and reusability were also investigated. L-Cyst-Fe_3_O_4_ NPs can be considered as fast, efficient, and biocompatible nano-adsorbent for Cr^6+^ and Pb^2+^ removal from contaminated waters.

## Electronic supplementary material

Supplementry Information
