# Supplementary material for: Lead and Chromium Adsorption from Water using L-Cysteine Functionalized Magnetite (Fe3O4) Nanoparticles
Source: Sci Rep. 2017 Aug 9;7:7672. doi: 10.1038/s41598-017-03380-x (PMC5550514; doi:10.1038/s41598-017-03380-x)
Supplement: Supplementary file 1 — Supplementry Information [file 41598_2017_3380_MOESM1_ESM.doc]

#### Supplementary Data

#### Lead and Chromium Adsorption from Water using

#### L-Cysteine Functionalized Magnetite (Fe_3_O_4_) Nanoparticles

#### Yana Bagbi^1, 3^, Ankur Sarswat^2^, Dinesh Mohan^2^, Arvind Pandey^3^, Pratima R. Solanki^1*^

^1^Special Centre for Nanoscience, Jawaharlal Nehru University, New Delhi-110067, India

^2^School of Environmental Sciences, Jawaharlal Nehru University, New Delhi-110067, India

^3^Department of Physics, North Eastern Regional Institute of Science and Technology, Nirjuli Arunachal Pradesh-791109, India

**Figure S1: Reaction mechanism for the formation of L-Cyst-Fe_3_O_4_ NPs.**

**
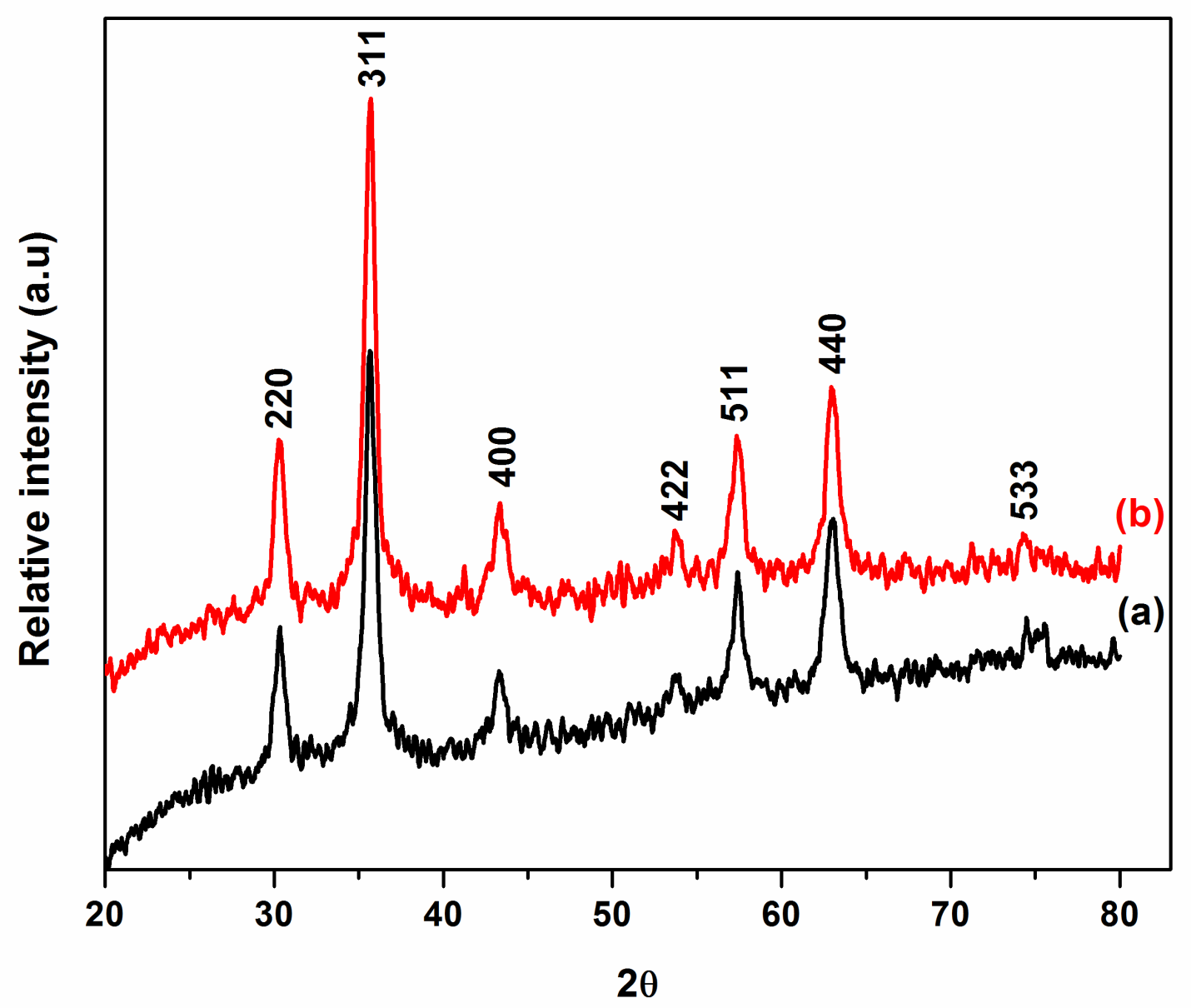
**

**Figure S2: Powder XRD patterns of (a) Fe_3_O_4_ and (b) L-Cyst- Fe_3_O_4_ NPs.**

**

**

**Figure S3: TEM micrographs of (a) Fe_3_O_4_ and (b) L-Cyst-Fe_3_O_4_ NPs at 10 kx magnification.**

**
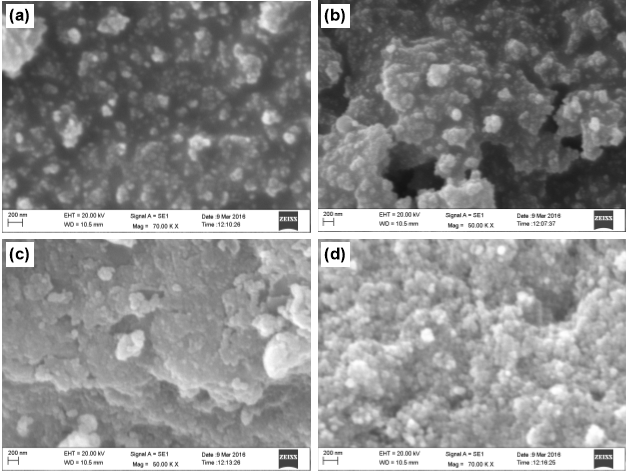
**

**Figure S4: SEM micrographs of Fe_3_O_4_ at (a) 70 kx and (b) 50 kx and L-Cyst-Fe_3_O_4_ NPs at (c) 50 kx and (d) 70 kx magnifications.**

**
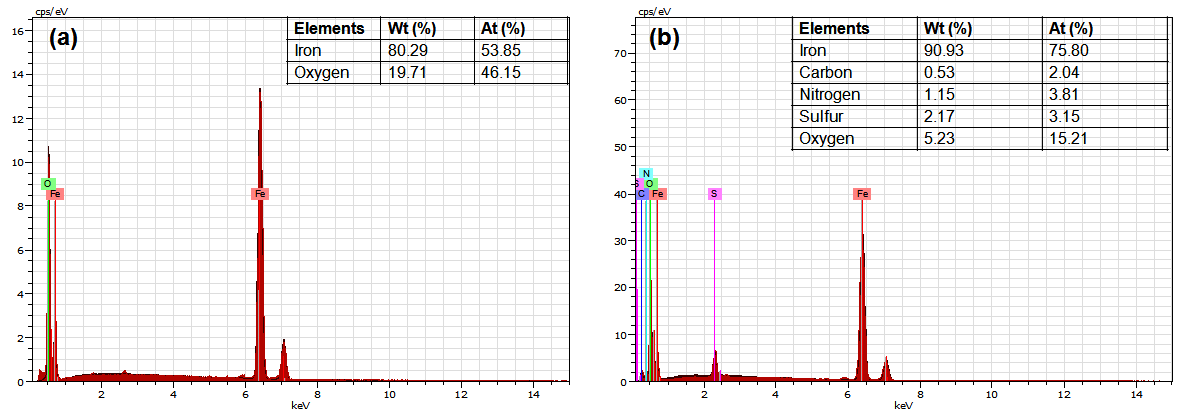
Figure S5: SEM-EDX spectra of (a) Fe_3_O_4_ and (b) L-Cyst-Fe_3_O_4_ NPs.**

**Figure S6: (i) UV-scan of (a) Fe_3_O_4_ and (b) L-Cyst-Fe_3_O_4_ NPs and (ii) Energy band gap plot of Fe_3_O_4_ and L-Cyst-Fe_3_O_4_ NPs.**

**
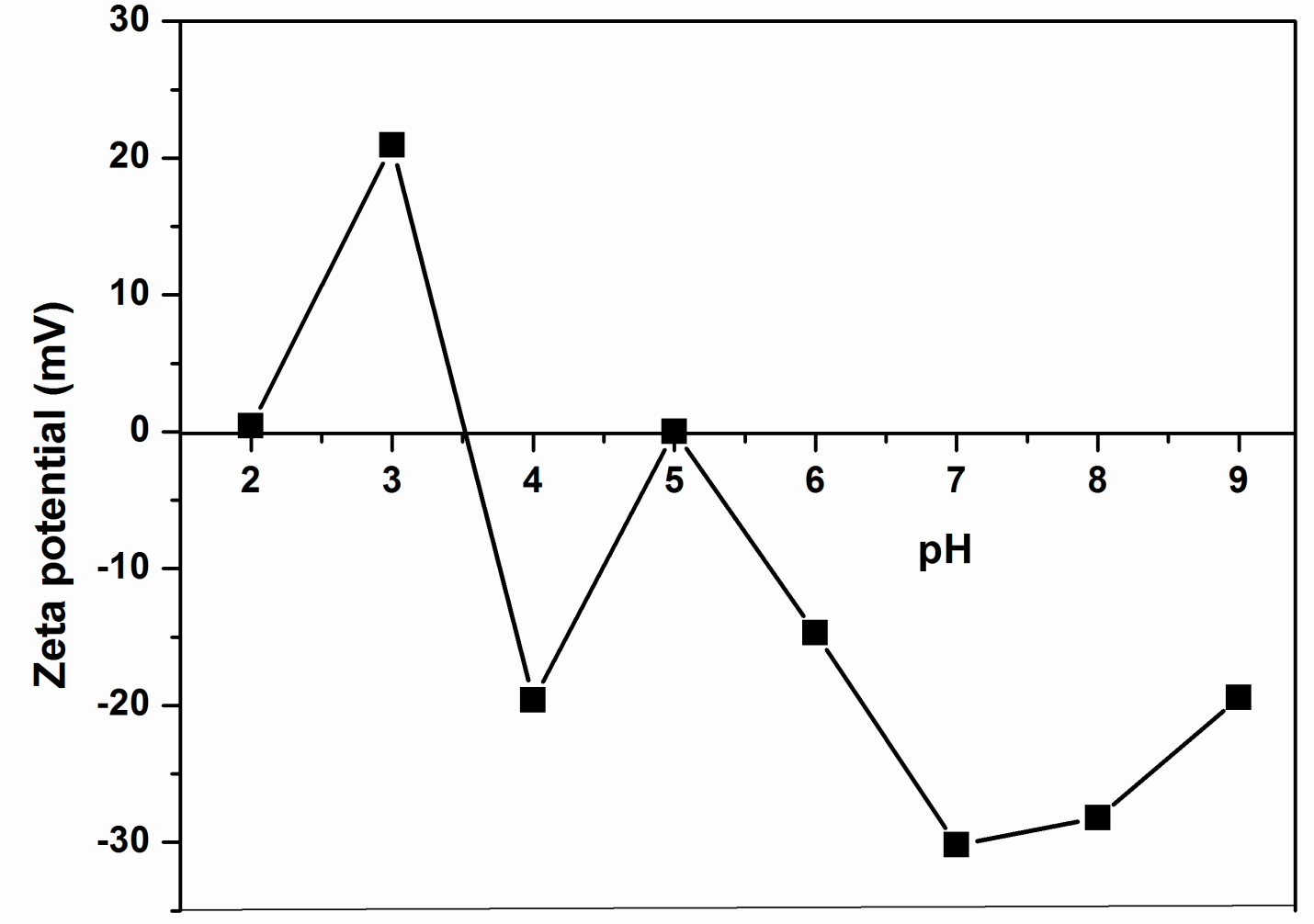
**

**Figure S7: Zeta potential of L-Cyst-Fe_3_O_4_ NPs at different aqueous pH varies from 2 to 9, dispersed at 20 mL of distilled water.**

**Figure S8: Langmuir separation factor (R_L_) for (a) Pb^2+^ and (b) Cr^6+^ adsorption on L-Cyst-Fe_3_O_4_ NPs at different temperatures.**

**
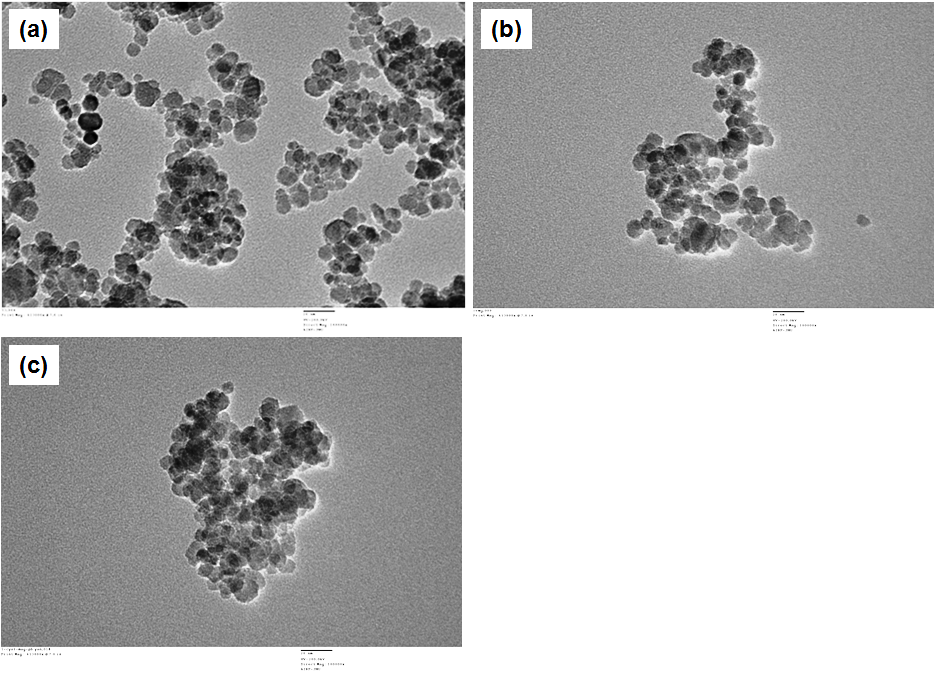
**

**Figure S9: TEM micrographs of L-Cyst-Fe_3_O_4_ NPs (a) before; (b) after five cycle’s chromium adsorption-desorption cycles and (c) five cycles lead adsorption-desorption cycles at 10 kx magnifications.**

**Table S1: N_2_ adsorption-desorption data of L-Cyst-Fe_3_O_4_ and Fe_3_O_4_ NPs.**

| **Adsorbent properties** | **L-Cyst-Fe_3_O_4_ NPs** | **Fe_3_O_4_ NPs** |
| --- | --- | --- |
| S_BET_ (m^2^g^-1^) | 58.49 | 75.69 |
| BJH Adsorption cumulative surface area of pores (m^2^/g) | 63.54 | 92.47 |
| BJH Desorption cumulative surface area of pores (m^2^/g) | 76.74 | 41.38 |
| BJH Adsorption cumulative volume of pores (cm^3^/g) | 0.12 | 0.24 |
| BJH Desorption cumulative volume of pores (cm^3^/g) | 0.12 | 0.17 |
| Adsorption total pore volume (cm^3^/g) | 0.16 | 0.22 |
| BJH Adsorption average Pore diameter (Å) | 74.81 | 107.51 |
| BJH Desorption average Pore diameter (Å) | 62.66 | 168.37 |
| Adsorption Average pore width (Å) | 108.94 | 119.20 |

**Table S2: Langmuir adsorption capacities of L-Cyst-Fe_3_O_4_ NPs versus other nanosorbents for Pb^2+^ and Cr^6+^**.

| **Nanoadsorbent** | **Temp. (° C)** | **Langmuir adsorption capacity (mg/g)** | | **References** |
| --- | --- | --- | --- | --- |
|  |  | **Pb^2+^** | **Cr^6+^** |  |
| L-Cyst-Fe_3_O_4_ NPs | 25 | 6.8 | 11.6 | Present study |
|  | 35 | 11.5 | 23.4 |  |
|  | 45 | 18.7 | 34.4 |  |
| Magnetite nanospheres | 25 | 13.4 | 6.6 | [40] |
|  | 35 | 14.1 | 7.3 |  |
|  | 45 | 18.4 | 8.9 |  |
| Ceria hollow nanospheres | - | 9.2 | - | [61] |
| SiO_2_/(3-aminopropyl) trimethoxysilane-coated magnetite NPs | - | 14.7 | - | [60] |
| ZnO nanocomposite | - | 6.7 | - | [68] |
| Modiﬁed jacobsite (MnFe_2_O_4_) NPs | - | - | 31.6 | [63] |
| Flowerlike α-Fe_2_O_3_ | - | - | 4.5 | [64] |
| Amino-modiﬁed Fe_3_O_4_ NPs | - | - | 11.2 | [65] |
| Fe_3_O_4_-γ-Fe_2_O_3_ NPs | - | - | 6.0 | [66] |
| Fe_3_O_4_/bacterial cellulose nanocomposite | - | - | 25.0 | [67] |
